# Piperidine-1-carboximidamide

**DOI:** 10.1107/S1600536812044467

**Published:** 2012-10-31

**Authors:** Ioannis Tiritiris

**Affiliations:** a Fakultät Chemie/Organische Chemie, Hochschule Aalen, Beethovenstrasse 1, D-73430 Aalen, Germany

## Abstract

In the title compound, C_6_H_13_N_3_, the C=N and C—N bond lengths in the CN_3_ unit are 1.3090 (17), and 1.3640 (17) (C–NH_2_) and 1.3773 (16) Å, indicating double- and single-bond character, respectively. The N—C—N angles are 116.82 (12), 119.08 (11) and 124.09 (11)°, showing a deviation of the CN_3_ plane from an ideal trigonal–planar geometry. The piperidine ring is in a chair conformation. In the crystal, mol­ecules are linked by N—H⋯N hydrogen bonds, forming a two-dimensional network along the *ac* plane.

## Related literature

For the crystal structure of 4-morpholine­carboxamidine, see: Tiritiris (2012 ▶). For the crystal structure of bis­(piperidin-1-yl)methanone, see: Betz *et al.* (2011 ▶).
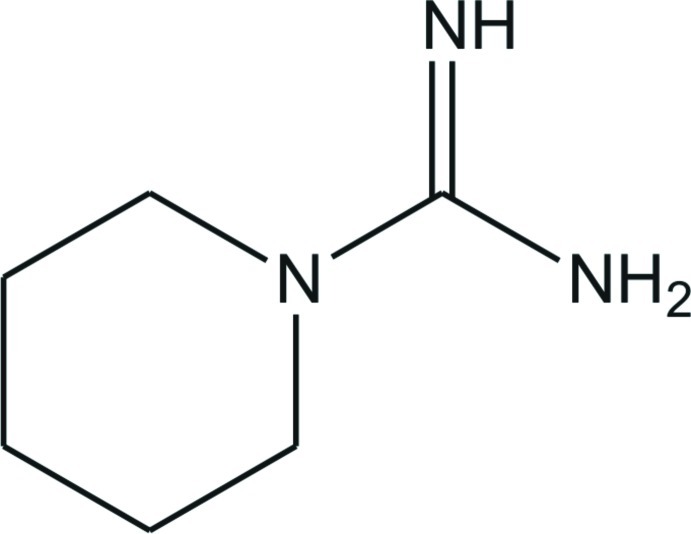


## Experimental

### 

#### Crystal data

C_6_H_13_N_3_
*M* *_r_* = 127.19
Monoclinic, 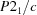

*a* = 12.2193 (9) Å
*b* = 5.5784 (5) Å
*c* = 10.4885 (7) Å
β = 91.887 (4)°
*V* = 714.55 (10) Å^3^
*Z* = 4
Cu *K*α radiation
μ = 0.60 mm^−1^
*T* = 100 K
0.45 × 0.26 × 0.06 mm

#### Data collection

Bruker Kappa APEXII DUO diffractometer
Absorption correction: multi-scan (Blessing, 1995 ▶) *T*_min_ = 0.830, *T*_max_ = 0.965
4190 measured reflections
1413 independent reflections
1116 reflections with *I* > 2σ(*I*)
*R*_int_ = 0.049

#### Refinement

*R*[*F* ^2^ > 2σ(*F* ^2^)] = 0.045
*wR*(*F* ^2^) = 0.112
*S* = 1.03
1413 reflections
94 parameters
H atoms treated by a mixture of independent and constrained refinement
Δρ_max_ = 0.19 e Å^−3^
Δρ_min_ = −0.21 e Å^−3^

### 

Data collection: *APEX2* (Bruker, 2008 ▶); cell refinement: *SAINT* (Bruker, 2008 ▶); data reduction: *SAINT*; program(s) used to solve structure: *SHELXS97* (Sheldrick, 2008 ▶); program(s) used to refine structure: *SHELXL97* (Sheldrick, 2008 ▶); molecular graphics: *DIAMOND* (Brandenburg & Putz, 2005 ▶); software used to prepare material for publication: *SHELXL97*.

## Supplementary Material

Crystal structure: contains datablock(s) I, global. DOI: 10.1107/S1600536812044467/go2073sup1.cif

Structure factors: contains datablock(s) I. DOI: 10.1107/S1600536812044467/go2073Isup2.hkl

Supplementary material file. DOI: 10.1107/S1600536812044467/go2073Isup3.cml

Additional supplementary materials:  crystallographic information; 3D view; checkCIF report

## Figures and Tables

**Table 1:** Hydrogen-bond geometry (Å, °)

*D*—H⋯*A*	*D*—H	H⋯*A*	*D*⋯*A*	*D*—H⋯*A*
N2—H21⋯N1^i^	0.94 (2)	2.15 (2)	3.071 (1)	168 (1)
N2—H22⋯N1^ii^	0.94 (2)	2.15 (2)	3.090 (1)	177 (1)

Symmetry codes: (i) 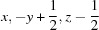; (ii) 
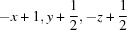
.

**Fig. 1.**
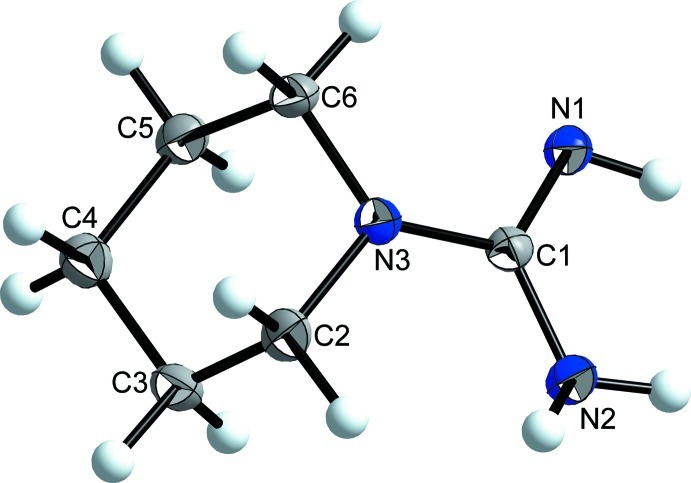
Molecular structure of the title compound with displacement ellipsoids at the 50% probability level.

**Fig. 2.**
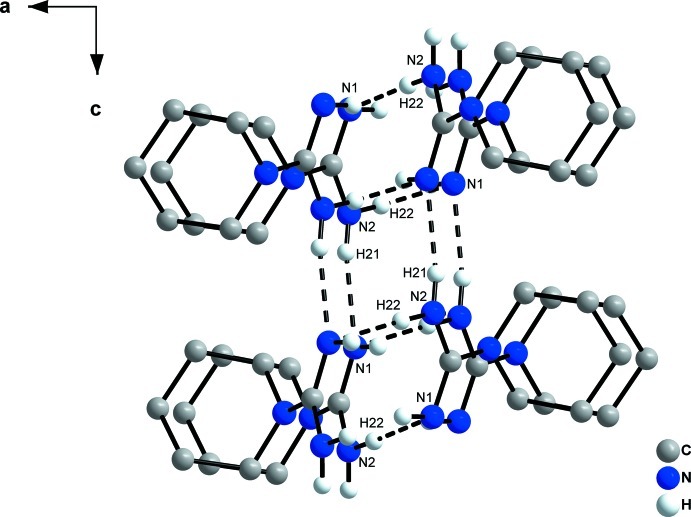
N–H···N hydrogen bonds between neighboring molecules, ac-view. The hydrogen bonds are indicated by dashed lines.

**Fig. 3.**
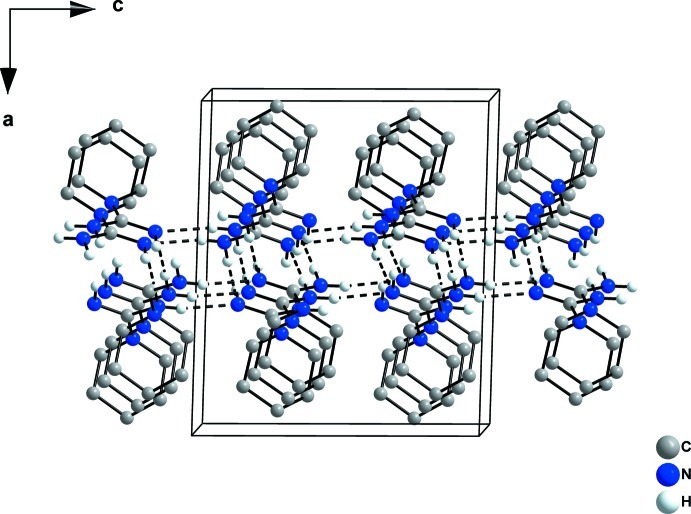
N–H···N hydrogen bonds generating a two-dimensional network, ac-view. The hydrogen bonds are indicated by dashed lines.

## supplementary crystallographic information

### Comment

1-Piperidinecarboxamidine, a guanidine derivative bearing one piperidine moiety, is similar to the structurally known compound 4-morpholinecarboxamidine (Tiritiris, 2012). Our efforts to study guanidines for CO_2_ capturing, led to the preparation of the title compound. Because its crystal structure was previously unknown, it was decided to conduct an investigation. According to the structure analysis, the C1–N1 bond in the title compound is 1.3090 (17) Å, indicating double bond character. The bond lengths C1–N2 = 1.3640 (17) Å and C1–N3 = 1.3773 (16) Å are elongated and characteristic for a C–N amine single bond (Fig. 1). The N–C1–N angles are: 116.82 (12)° (N2–C1–N3), 119.08 (11)° (N1–C1–N3) and 124.09 (11)° (N1–C1–N2), showing a deviation of the CN_3_ plane from an ideal trigonal-planar geometry (Fig. 1). The structural parameters of the piperidine ring in the here presented title compound agree very well with the data obtained from the X-ray analysis of the urea bis(piperidin-1-yl)methanone (Betz *et al.*, 2011). In both crystal structures the piperidine rings adopt a chair conformation. In contrast to the structure of 4-morpholinecarboxamidine (Tiritiris, 2012), only strong N—H···N hydrogen bonds between nitrogen atoms of neighboring molecules (Fig. 2 and 3) are present [*d*(H···N) = 2.15 (2) Å] (Tab. 1), forming an infinite two-dimensional network (base vectors [0 0 1] and [0 1 0]). Surprisingly, the imine hydrogen atom H11 is not involved in the hydrogen bonding system.

### Experimental

1-Piperidine-carboxamidinium sulfate (I) was prepared by heating one equivalent *O*-methylisourea sulfate with two equivalents of piperidine under reflux. The methanol formed in the reaction was distilled off and (I) precipitated in nearly quantitative yield. To a solution of 5.0 g (14 mmol) (I) in 50 ml water, a solution of 1.2 g (30 mmol) sodium hydroxide dissolved in 25 ml water was added dropwise under ice cooling. After warming to room temperature the aqueous phase was extracted with diethyl ether. The organic phase was finally dried over sodium sulfate. After evaporation of the solvent, the title compound precipitated in form of a colorless solid. Yield: 1.5 g (84%). During the storage of a saturated acetonitrile solution at 0° C, colorless single crystals of the title compound suitable for X-ray analysis were obtained. ^1^H NMR (500 MHz, CD_3_CN/TMS): δ = 1.60–1.64 [m, 6 H, –CH_2_], 3.38–3.42 [m, 4 H,–CH_2_], 5.85 [s, 1 H, –NH], 6.19 [s, 2 H, –NH_2_]. ^13^C NMR (125 MHz, CD_3_CN/TMS): δ = 23.2 (–CH_2_), 24.7 (–CH_2_), 46.5 (–CH_2_), 157.4 (C═N).

### Refinement

The N-bound H atoms were located in a difference Fourier map and were refined freely [N—H = 0.91 (2)–0.94 (2) Å]. The hydrogen atoms of the methylene groups were placed in calculated positions with d(C—H) = 0.99 Å. They were included in the refinement in the riding model approximation, with *U*(H) set to 1.2 *U*_eq_(C).

### Crystal data

**Table:**

C_6_H_13_N_3_	*F*(000) = 280
*M**_r_* = 127.19	*D*_x_ = 1.182 Mg m^−^^3^
Monoclinic, *P*2_1_/*c*	Melting point: 409 K
Hall symbol: -P 2ybc	Cu *K*α radiation, λ = 1.54178 Å
*a* = 12.2193 (9) Å	Cell parameters from 4190 reflections
*b* = 5.5784 (5) Å	θ = 3.6–73.5°
*c* = 10.4885 (7) Å	µ = 0.60 mm^−^^1^
β = 91.887 (4)°	*T* = 100 K
*V* = 714.55 (10) Å^3^	Plate, colorless
*Z* = 4	0.45 × 0.26 × 0.06 mm

### Data collection

**Table:**

Bruker Kappa APEXII DUO diffractometer	1413 independent reflections
Radiation source: sealed tube	1116 reflections with *I* > 2σ(*I*)
Graphite monochromator	*R*_int_ = 0.049
φ scans, and ω scans	θ_max_ = 73.5°, θ_min_ = 3.6°
Absorption correction: multi-scan (Blessing, 1995)	*h* = −15→15
*T*_min_ = 0.830, *T*_max_ = 0.965	*k* = −6→6
4190 measured reflections	*l* = −12→12

### Refinement

**Table:**

Refinement on *F*^2^	Primary atom site location: structure-invariant direct methods
Least-squares matrix: full	Secondary atom site location: difference Fourier map
*R*[*F*^2^ > 2σ(*F*^2^)] = 0.045	Hydrogen site location: difference Fourier map
*wR*(*F*^2^) = 0.112	H atoms treated by a mixture of independent and constrained refinement
*S* = 1.03	*w* = 1/[σ^2^(*F*_o_^2^) + (0.0625*P*)^2^] where *P* = (*F*_o_^2^ + 2*F*_c_^2^)/3
1413 reflections	(Δ/σ)_max_ < 0.001
94 parameters	Δρ_max_ = 0.19 e Å^−^^3^
0 restraints	Δρ_min_ = −0.21 e Å^−^^3^

### Special details

**Table:**

Geometry. All e.s.d.'s (except the e.s.d. in the dihedral angle between two l.s. planes) are estimated using the full covariance matrix. The cell e.s.d.'s are taken into account individually in the estimation of e.s.d.'s in distances, angles and torsion angles; correlations between e.s.d.'s in cell parameters are only used when they are defined by crystal symmetry. An approximate (isotropic) treatment of cell e.s.d.'s is used for estimating e.s.d.'s involving l.s. planes.
Refinement. Refinement of *F*^2^ against ALL reflections. The weighted *R*-factor *wR* and goodness of fit *S* are based on *F*^2^, conventional *R*-factors *R* are based on *F*, with *F* set to zero for negative *F*^2^. The threshold expression of *F*^2^ > σ(*F*^2^) is used only for calculating *R*-factors(gt) *etc*. and is not relevant to the choice of reflections for refinement. *R*-factors based on *F*^2^ are statistically about twice as large as those based on *F*, and *R*- factors based on ALL data will be even larger.

### Fractional atomic coordinates and isotropic or equivalent isotropic displacement parameters (Å^2^)

**Table:**

	*x*	*y*	*z*	*U*_iso_*/*U*_eq_	
C1	0.38570 (11)	0.2272 (2)	0.20721 (12)	0.0194 (3)	
N1	0.41982 (10)	0.26476 (18)	0.32513 (11)	0.0229 (3)	
H11	0.4665 (14)	0.393 (3)	0.3256 (16)	0.031 (4)*	
N2	0.40843 (11)	0.37432 (19)	0.10763 (12)	0.0248 (3)	
H21	0.4040 (14)	0.315 (3)	0.0240 (18)	0.037 (4)*	
H22	0.4598 (14)	0.497 (3)	0.1251 (15)	0.035 (4)*	
N3	0.32466 (10)	0.02529 (17)	0.17918 (10)	0.0225 (3)	
C2	0.25890 (12)	−0.0007 (2)	0.06138 (13)	0.0270 (4)	
H2A	0.2591	−0.1706	0.0341	0.032*	
H2B	0.2915	0.0964	−0.0067	0.032*	
C3	0.14128 (12)	0.0807 (2)	0.08029 (14)	0.0279 (4)	
H3A	0.1403	0.2540	0.1006	0.034*	
H3B	0.0972	0.0553	0.0005	0.034*	
C4	0.09150 (13)	−0.0602 (2)	0.18847 (14)	0.0275 (3)	
H4A	0.0179	0.0040	0.2054	0.033*	
H4B	0.0833	−0.2304	0.1631	0.033*	
C5	0.16364 (12)	−0.0434 (2)	0.30906 (14)	0.0273 (4)	
H5A	0.1341	−0.1493	0.3754	0.033*	
H5B	0.1629	0.1231	0.3416	0.033*	
C6	0.28172 (12)	−0.1171 (2)	0.28289 (13)	0.0246 (3)	
H6A	0.3282	−0.0937	0.3610	0.030*	
H6B	0.2837	−0.2892	0.2600	0.030*	

### Atomic displacement parameters (Å^2^)

**Table:**

	*U*^11^	*U*^22^	*U*^33^	*U*^12^	*U*^13^	*U*^23^
C1	0.0203 (7)	0.0167 (6)	0.0211 (7)	0.0021 (4)	0.0004 (5)	−0.0011 (4)
N1	0.0271 (7)	0.0187 (5)	0.0228 (6)	−0.0023 (4)	−0.0013 (5)	−0.0015 (4)
N2	0.0335 (7)	0.0211 (5)	0.0196 (6)	−0.0053 (5)	−0.0011 (5)	−0.0009 (4)
N3	0.0256 (7)	0.0203 (5)	0.0214 (6)	−0.0032 (4)	−0.0036 (5)	0.0001 (4)
C2	0.0329 (9)	0.0261 (6)	0.0218 (7)	−0.0065 (5)	−0.0021 (6)	−0.0037 (5)
C3	0.0309 (9)	0.0274 (6)	0.0249 (8)	−0.0016 (6)	−0.0091 (6)	0.0017 (5)
C4	0.0261 (8)	0.0268 (7)	0.0295 (8)	0.0009 (5)	−0.0013 (6)	−0.0002 (5)
C5	0.0313 (9)	0.0251 (6)	0.0254 (8)	−0.0022 (5)	0.0012 (6)	0.0016 (5)
C6	0.0292 (8)	0.0197 (6)	0.0247 (7)	−0.0019 (5)	−0.0040 (6)	0.0048 (5)

### Geometric parameters (Å, º)

**Table:**

C1—N1	1.3090 (17)	C3—C4	1.5235 (19)
C1—N2	1.3640 (17)	C3—H3A	0.9900
C1—N3	1.3773 (16)	C3—H3B	0.9900
N1—H11	0.913 (17)	C4—C5	1.521 (2)
N2—H21	0.937 (18)	C4—H4A	0.9900
N2—H22	0.943 (17)	C4—H4B	0.9900
N3—C6	1.4585 (17)	C5—C6	1.534 (2)
N3—C2	1.4587 (17)	C5—H5A	0.9900
C2—C3	1.526 (2)	C5—H5B	0.9900
C2—H2A	0.9900	C6—H6A	0.9900
C2—H2B	0.9900	C6—H6B	0.9900
			
N1—C1—N2	124.09 (11)	C2—C3—H3B	109.6
N1—C1—N3	119.08 (11)	H3A—C3—H3B	108.2
N2—C1—N3	116.82 (12)	C5—C4—C3	110.67 (12)
C1—N1—H11	108.1 (11)	C5—C4—H4A	109.5
C1—N2—H21	119.8 (10)	C3—C4—H4A	109.5
C1—N2—H22	116.1 (10)	C5—C4—H4B	109.5
H21—N2—H22	117.2 (14)	C3—C4—H4B	109.5
C1—N3—C6	119.46 (11)	H4A—C4—H4B	108.1
C1—N3—C2	122.78 (10)	C4—C5—C6	110.95 (12)
C6—N3—C2	112.09 (10)	C4—C5—H5A	109.4
N3—C2—C3	110.80 (11)	C6—C5—H5A	109.4
N3—C2—H2A	109.5	C4—C5—H5B	109.4
C3—C2—H2A	109.5	C6—C5—H5B	109.4
N3—C2—H2B	109.5	H5A—C5—H5B	108.0
C3—C2—H2B	109.5	N3—C6—C5	110.55 (11)
H2A—C2—H2B	108.1	N3—C6—H6A	109.5
C4—C3—C2	110.12 (11)	C5—C6—H6A	109.5
C4—C3—H3A	109.6	N3—C6—H6B	109.5
C2—C3—H3A	109.6	C5—C6—H6B	109.5
C4—C3—H3B	109.6	H6A—C6—H6B	108.1
			
N1—C1—N3—C6	11.63 (18)	N3—C2—C3—C4	−56.89 (14)
N2—C1—N3—C6	−169.57 (11)	C2—C3—C4—C5	53.91 (15)
N1—C1—N3—C2	162.94 (12)	C3—C4—C5—C6	−53.30 (14)
N2—C1—N3—C2	−18.25 (18)	C1—N3—C6—C5	95.35 (14)
C1—N3—C2—C3	−93.09 (14)	C2—N3—C6—C5	−58.83 (14)
C6—N3—C2—C3	60.10 (13)	C4—C5—C6—N3	55.17 (13)

### Hydrogen-bond geometry (Å, º)

**Table:**

*D*—H···*A*	*D*—H	H···*A*	*D*···*A*	*D*—H···*A*
N2—H21···N1^i^	0.94 (2)	2.15 (2)	3.071 (1)	168 (1)
N2—H22···N1^ii^	0.94 (2)	2.15 (2)	3.090 (1)	177 (1)

Symmetry codes: (i) *x*, −*y*+1/2, *z*−1/2; (ii) −*x*+1, *y*+1/2, −*z*+1/2.
